# Cardiorespiratory arrest during and after nuss procedure: case report

**DOI:** 10.1186/s13019-023-02262-w

**Published:** 2023-04-28

**Authors:** Indira F. Cujiño-Álvarez, Daniela Torres-Salazar, Mauricio Velásquez-Galvis

**Affiliations:** 1 Anesthesiology Department, Anesthestiologist. Fundación Valle del Lili, Cra 98 No. 18-49, Cali, 760032 Colombia; 2 Anesthesiology Resident. Universidad Icesi, Facultad de Ciencias de la Salud, Calle 18 No. 122- 135, Cali, Colombia; 3 Thoracic Surgery Department, Thoracic Surgeon. Fundación Valle del Lili, Cra 98 No. 18-49, Cali, 760032 Colombia

**Keywords:** Nuss procedure, Case report, Cardiopulmonary resuscitation, Pectum excavatum, Postoperative complications

## Abstract

**Background:**

Pectum excavatum is a congenital thoracic alteration that can present important physiological alterations depending on the severity of the case. The Nuss procedure is a minimally invasive technique for managing chest wall deformity, in which there is a risk of perioperative complications.

**Case presentation:**

This article presents the case of a 16-year-old patient who underwent placement of a Nuss bar and suffered intraoperative and postoperative cardiorespiratory arrest.

**Conclusions:**

it is important to consider the possible early and late complications scenarios as well as their treatment in patients with pectum excavatum scheduled for a Nuss procedure.

## Background

Pectum excavatum (PE) is a congenital thoracic disorder with an incidence of 1 per 400 births and a prevalence of 2.6% in children aged 7 to 14 years [1]. This deformity predominantly affects men, at a ratio of 5:1 with women [2]. Depending on its severity, it can generate some physiological changes, such as decreased cardiac function, restriction of lung function, and psychological sequelae due to the physical appearance of the patient [3]. Different techniques have been developed to repair this pathology. One of them, the Nuss procedure, was described for the first time in 1998 as a minimally invasive technique that consists of the placement of a metal bar underneath the sternum to reshape the anterior chest wall [4]. Perioperative complications of the Nuss procedure are rare but can threaten the life of the patient [5–8]. It is not clear how cardiopulmonary resuscitation (CPR) should be performed in patients with a Nuss bar, since the bar can generate technical difficulties for chest compressions and defibrillation [9–11].

We present the case of a 16-year-old patient who had intraoperative cardiorespiratory arrest during a Nuss procedure and postoperatively in the intensive care unit.

## Case presentation

**Fig. 1:**
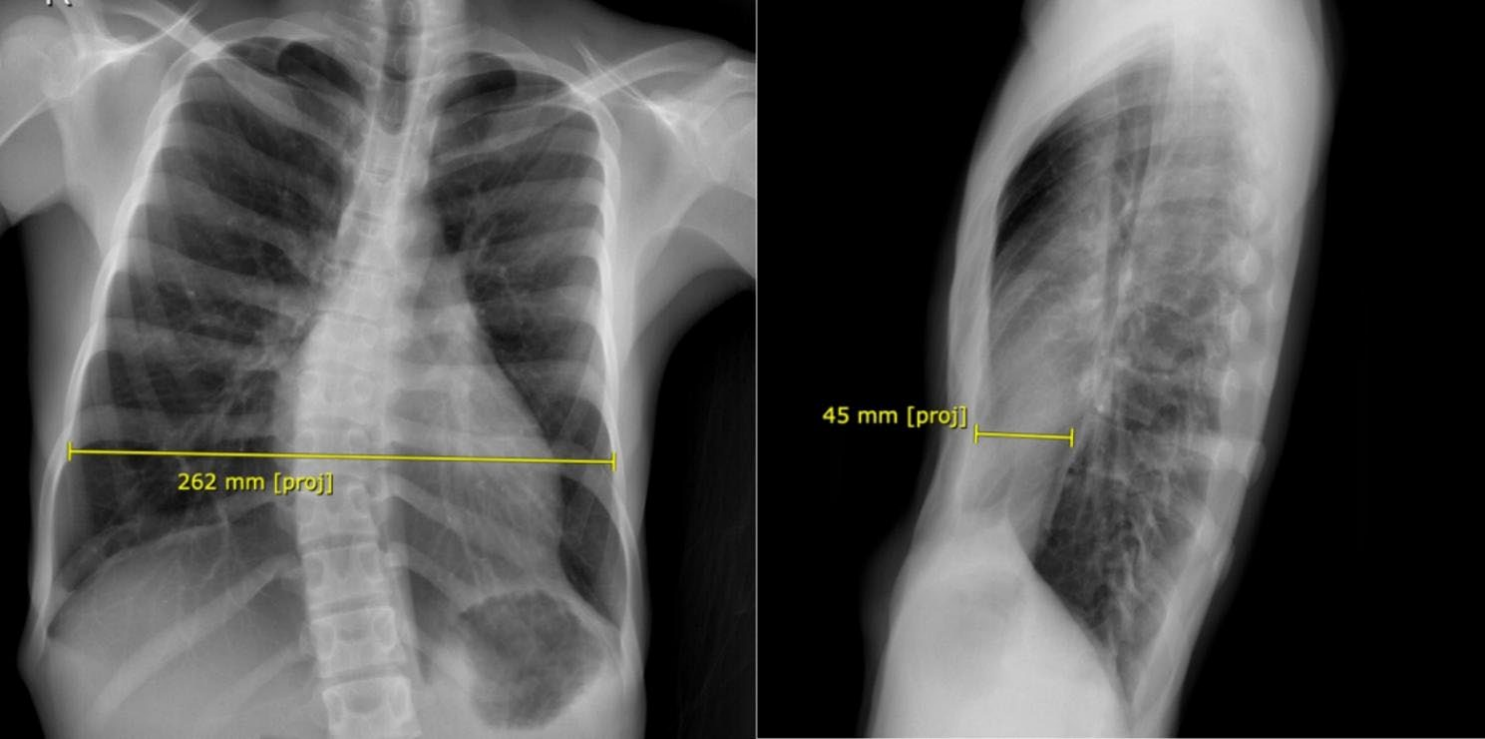
Preoperative chest X-ray. The transverse and anteroposterior diameters were measured with a Haller index of 5.8

A 16-year-old male patient with Eagle–Barrett syndrome and congenital pectum excavatum was admitted for surgical correction of chest deformity. The preoperative chest X-ray (Fig. 1) showed a Haller index of 5.8 and an electrocardiogram with sinus rhythm and right bundle branch block. The estimated functional capacity of the patient was greater than 6 metabolic equivalents (METs).

On September 6, 2018, a videothoracoscopy-guided Nuss procedure was performed. It was monitored according to the recommendations of the American Society of Anesthesiologists, and a thoracic epidural catheter was placed at the T4-T5 level for postoperative analgesia without complications. Intravenous anesthesia and placement of a left double-lumen endotracheal tube No. 35 followed.

During the passage of the Nuss bar, the patient presented episodes of ventricular tachycardia with a pulse that subsided when the surgical stimulus was suspended. The passage of the bar was continued, and after turning it to correct the defect and leaving it in position, the patient presented severe bradycardia that improved with atropine. Two minutes after having the bar fully in position and before starting to fix it with the stabilizer, he presented ventricular fibrillation. Cardiopulmonary resuscitation was initiated with chest compressions in the lower half of the sternum, placing the hands below the Nuss bar. Three external defibrillations were performed at 200 joules with the defibrillator paddles placed in a conventional manner (right infraclavicular area and cardiac apex). Additionally, two 1-mg intravenous boluses of epinephrine were administered 5 min apart.

Ten minutes after arrest, the patient returned to spontaneous circulation. The fibrillation presented was not continuous but came in two episodes, and in between them, we removed the Nuss bar. After the removal of the rod and with the patient in sinus rhythm, a left radial artery and a right internal jugular central venous catheter were channeled, guided by ultrasound, without complications. The patient continued to have hemodynamic instability, so norepinephrine infusion was started at 0.1 µg/kg/min, with an adequate response. The thoracoscope was reintroduced, and the heart was checked for structural lesions.

A transesophageal echocardiography probe was inserted to verify that there were no cardiac lesions and was used as a guide for volume resuscitation. Blood samples were taken to check the metabolic status of the patient and look for other causes that explained his cardiac arrest.

**Fig. 2:**
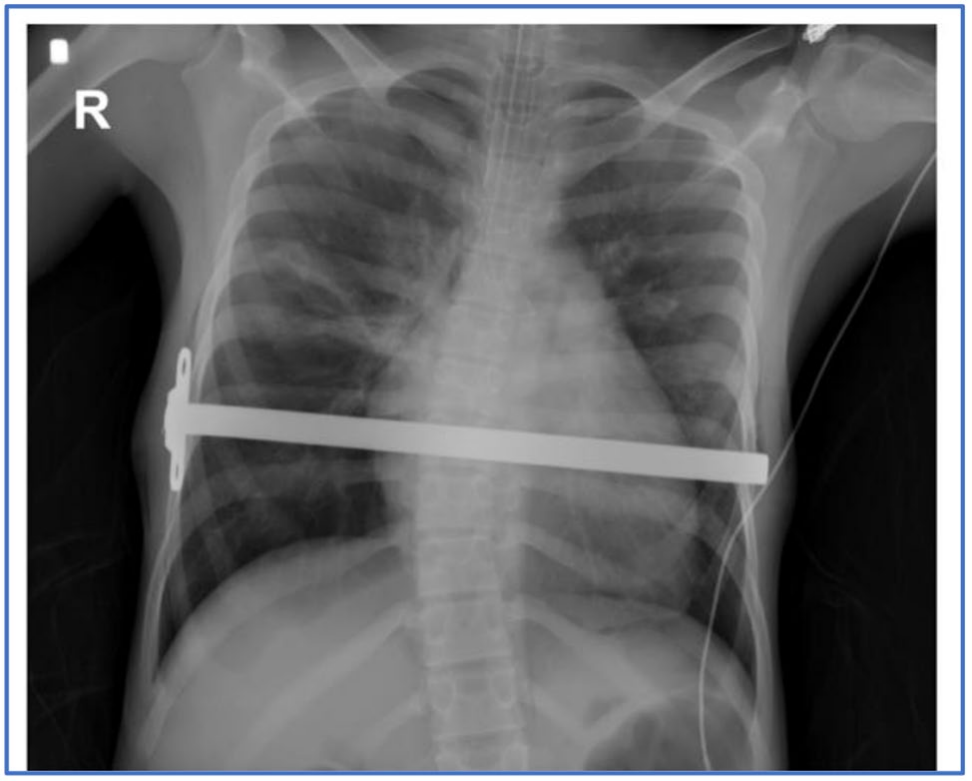
Intraoperative chest X ray after Nuss bar insertion

Since the patient presented significant improvement with a decrease in the use of vasopressors and persistence of sinus rhythm, it was decided to pass the Nuss bar again without finding any alteration of the heart rhythm or hemodynamic changes. The procedure was completed without any other complications. Chest X-ray (Fig. 2) showed a small right pneumothorax without any indication for invasive management.

At the end of the surgery, the neuromuscular blockade was reversed with sugammadex and was confirmed with a train of four test result > 95%. The patient was extubated in the operating room to assess his neurological status. He could move all four limbs, had symmetrical 3-mm pupils with adequate reactivity to light, was alert, and obeyed orders. He was transferred to the pediatric intensive care unit, where he was given oxygen through a 50% Venturi mask.

Hours later, in the intensive care unit, a new chest X-ray showed significant bilateral pneumothorax, for which they performed bilateral thoracostomies. A transthoracic echocardiogram did not show structural cardiac damage, but there was dysfunction of the left ventricle with decreased segmental and global contractility and a left ventricular ejection fraction of 45%, without other abnormalities.

During the first postoperative day, the patient persisted with hemodynamic instability that required vasoactive and inotropic support. At approximately 20 h postoperatively, he presented cardiorespiratory arrest with pulseless electrical activity on two occasions for 10 min each. Chest compressions were performed on the lower half of the sternum, and intravenous calcium gluconate, sodium bicarbonate, and epinephrine were administered. The patient continued with hemodynamic instability and the need for vasoactive and inotropic support in increasing doses.

The medical team decided to remove the Nuss bar, which was achieved without complications and with good postoperative evolution. The control echocardiogram the day after the rod was removed showed adequate biventricular function and a left ventricular ejection fraction of 80%. The next day, he underwent successful extubation. After progressive withdrawal of hemodynamic support, he was discharged from the hospital 12 days after surgery. The patient has been evaluated with ambulatory controls without any alterations of note. One year after this complication, he presented good and stable functional and psychological capacity.

## Discussion

Performing the Nuss procedure has become the most widely used technique for the correction of PE in pediatric and adult populations [3, 4]. This procedure brings the risk of lethal complications, although rare, such as lung, heart, or great vessel injury [12, 13]. Major and minor complications have been reported with an incidence that varies between 2% and 20%, although the true incidence is unknown due to the underreporting of these cases [7, 8, 12, 13].

In the literature, almost all published Nuss procedure related cardiac arrests are caused by cardiac or vascular injury. Interestingly in this case the cardiac arrest was not caused by physical heart or vessel damage. There are two possible mechanisms for this rare complication reported in the literature [8]: the elevation of the sternum may have caused slightly rotation of the heart and the great vessels with myocardial ischemia and consequent ventricular fibrillation. And the sudden enlargement of the retrosternal space may have caused a nerve stretch reflex similar to the trigeminocardiac reflex which might have upset the balance between vagal and sympathetic innervation and triggered inhibition of cardiac function and consequent arrest [8].

In patients who present complications during the Nuss procedure that lead to cardiac arrest, advanced CPR is a source of controversy and a challenge for surgical teams since the placement of the Nuss bar makes it much harder to perform chest compressions. The Nuss bar impairs the excursion of the anterior chest wall, which is necessary during CPR maneuvers to achieve compression of the heart between the sternum and the spine. The guidelines of the American Heart Association (AHA) and the International Liaison Committee on Resuscitation (ILCOR) do not give specific recommendations on CPR in patients with a Nuss bar or PE, and the evidence that exists about CPR maneuvers in these patients is scant. In the case reports published to date, recommendations are given based on the clinical, biological, physiological, and imaging knowledge of these patients. They recommend applying compressions with a substantial increase in force, which should be greater the more bars the patient has [9]. In addition, in cases of intraoperative CPR, immediate removal of the metal bar and the performance of high-quality chest compressions are recommended [5].

Through an elastic model, the relationship between chest compression forces and chest displacement was simulated to achieve 5 cm of anterior wall mobility (recommendation of the AHA, as an objective parameter of compressions in CPR), and the presence of Nuss bars did not allow effective cardiopulmonary resuscitation [9, 14].

Active abdominal compression and decompression is a novel technique that appears to improve artificial circulation using thoracic, abdominal, and cardiac pumping mechanisms. This relatively new CPR method induces intra-abdominal pressure changes that activate the abdominal pump to transmit pressure to the thoracic cavity, which in turn activates the thoracic and cardiac pumps through the action of the diaphragm [15]. Although there are no reports on the use of active abdominal compression and decompression in CPR after PE repair, the findings suggest that this alternative method of CPR may have potential utility in these types of patients.

It is also important to determine the best places for external defibrillation paddles in a patient with a Nuss bar, taking into account that some complications occur with shockable arrest rhythms, as in the case of our patient. We positioned the defibrillation paddles based on the AHA and ILCOR guidelines. The usual position of the paddles can allow the flow of electricity from one paddle to the other, passing through the Nuss bar and without passing through the myocardium. Therefore, it is recommended to place the paddles as follows: one anterior on the sternal midline and the other posterior, placing it between the scapulae, forcing the current to pass through the heart [8].

Our recommendations to reduce and detect perioperative complications in these patients are as follows:

1. Take an electrocardiogram and echocardiogram before surgery, as proposed by Zou et al. in their review [8]. In some case reports, fatal complications of Nuss bar placement have been associated with structural or functional alterations of the heart.
2. Keep in mind the possibility of cardiac arrhythmia during the placement and removal of the Nuss bar, monitor the progression, and remove the bar promptly when they occur.
3. Perform intraoperative transesophageal echocardiography in cases of suspected complications to ensure that they are not secondary to direct trauma due to the passage of the bar or its compressive effect on cardiac structures.

## Conclusions

In patients with PE who are scheduled for a Nuss procedure, it is important to know the possible early and late complications, as well as the management of these according to the scenarios in which they arise. Future research should determine how best to manage chest compressions in patients with metal bars in the chest without the need to remove them.

## Data Availability

Data sharing is not applicable to this article as no datasets were generated or analysed during the current study.

## Abbreviations

PE: Pectum excavatum
CPR: Cardiopulmonary resuscitation
METs: Metabolic equivalents
AHA: American Heart Association
ILCOR: International Liaison Committee on Resuscitation
